# Do depression, anxiety, emotional intelligence, and sleep quality affect diabetes management self-efficacy in Korean women with gestational diabetes in pregnancy?: a descriptive correlational study

**DOI:** 10.4069/kjwhn.2021.11.27

**Published:** 2021-12-29

**Authors:** Hoon Ah Lee, Keum Seong Jang

**Affiliations:** 1 College of Nursing, Graduate School, Chonnam National University, Gwangju, Korea; 2 College of Nursing, Chonnam National University, Gwangju, Korea

**Keywords:** Emotional intelligence, Gestational diabetes, Self-efficacy, Sleep

## Abstract

**Purpose:**

This study aimed to identify factors associated with diabetes management self-efficacy in pregnant women with gestational diabetes mellitus (GDM) in Korea.

**Methods:**

A total of 173 pregnant women with GDM in Korea were recruited by posting announcements at two Korean online communities focusing on pregnancy and GDM. Participants completed a structured online survey from July to September 2018. Thirteen inappropriate responses were excluded and a total of 160 questionnaires were used in the final analysis. Descriptive statistics were calculated and multiple regression with the enter method was done to identify the associations of depressive mood, anxiety, emotional intelligence, and sleep quality with diabetes management self-efficacy.

**Results:**

Respondents reported a moderately depressive mood (mean, 10.36), low to moderate anxiety (mean, 41.65), above-average emotional intelligence (mean, 78.04), moderate sleep quality (mean, 42.01), and above-average diabetes management self-efficacy (mean, 52.29). The major factor associated with diabetes management self-efficacy of pregnant women with GDM was emotional intelligence (β=.51, *p*<.001). Other factors, in descending order of influence, were sleep quality (β=.22, *p*<.001) and exercise (β=.18, *p*=.004). Taken together, the aforementioned factors explained 34.6% (F=39.53, *p*<.001) of the total variance.

**Conclusion:**

The results of this study suggest that to improve the diabetes management self-efficacy of pregnant women with GDM, it is necessary to develop an education program that can also enhance emotional intelligence, sleep quality, and exercise.

## Introduction

Gestational diabetes mellitus (GDM) is diagnosed when glucose intolerance develops after 20 weeks of pregnancy in a woman who previously did not have diabetes [1]. According to a recent study, the incidence of GDM sharply increased by 30% in women aged 15 to 44 years who had their first live birth between 2011 and 2019, and the highest rate of increase was noted among women of Asian descent [2]. GDM increases the risk of perinatal complications [1]. According to a recent meta-analysis [3], compared to pregnant women without GDM, those with GDM were 8.3 times more likely to develop type 2 diabetes after childbirth, and approximately 17% had diabetes later in life. Thus, GDM is a gestational disease that requires lifestyle changes for lifelong management.

Self-efficacy in diabetes management reflects individual beliefs and refers to the patient’s degree of self-confidence that they can practice healthy behaviors and manage their diabetes. This confidence has been reported as the strongest influencing factor in diabetes self-management and healthy behavior practices [4]. However, pregnant women who are unexpectedly diagnosed with GDM experience diabetes self-management as a substantial burden because they have to be educated about diabetes in a short period of time and must now make efforts to improve their overall lifestyle behaviors.

Patients with diabetes who are in a negative psychological state experience more difficulty in implementing self-management due to impaired emotional strength [5]. Pregnant women with GDM experience a negative emotional state the moment they receive a diagnosis of diabetes during pregnancy [6]. Depression makes it more difficult for patients with diabetes to control their blood sugar levels; thus, along with impaired self-care, depression is a risk factor for blood sugar control failure [5].

Concerns about fetal health are the primary cause for anxiety in pregnant women and pregnant women with GDM experience greater anxiety than pregnant women without GDM [6]. The negative emotions of pregnant women with GDM can impair their ability to actively manage their diabetes in the already challenging journey through pregnancy [5,6]. However, there is a lack of research on the effect of anxiety and depression on diabetes management self-efficacy for pregnant women with GDM.

Emotional intelligence helps pregnant women with GDM maintain rational thinking even in a negative emotional state, helping them to empathize with others and maintain hope, to motivate themselves even in exhausting situations, and to control impulses for immediate gratification [7]. These benefits of emotional intelligence are relevant to diabetes management self-efficacy. A recent study showed that higher emotional intelligence was related to better blood sugar control [8]. However, no studies have explored the relationship between emotional intelligence and diabetes management self-efficacy in women with GDM.

The physical effects resulting from impaired sleep and poor sleep quality are experienced by people with negative emotions [9]. In a recent systematic review and meta-analysis, insufficient sleep duration and poor sleep quality were identified as significant risk factors for developing GDM [10]. Although the effect of poor sleep quality on diabetes has been proven by several previous epidemiological studies [11], there have been no studies on the direct impact of sleep quality on diabetes management self-efficacy among pregnant women with GDM.

Therefore, we hypothesized that diabetes management self-efficacy was crucial for pregnant women with GDM to successfully practice diabetes management, and that various psychological, emotional, and physical factors could influence that self-efficacy. However, the existing literature has been focused on the changing trends in the treatment of pregnant women with GDM [2,3], the influence of self-efficacy on improved treatment compliance in this population [4], and the level of improvement in self-management or blood sugar control after interventions [10]. To the authors’ knowledge, no studies have yet investigated the influence of the factors introduced above on the level of diabetes management self-efficacy.

Thus, the purpose of this study was to examine diabetes management self-efficacy among pregnant women with GDM and to assess the effects of depression, anxiety, emotional intelligence, and sleep quality on self-efficacy. The ultimate goal was to provide basic data for developing interventions that enhance diabetes management self-efficacy in pregnant women with GDM.

The specific objectives of this study were as follows: (1) to identify differences in diabetes management self-efficacy according to the general and clinical characteristics of pregnant women with GDM; (2) to assess the degree of depression, anxiety, emotional intelligence, sleep quality, and diabetes management self-efficacy; (3) to evaluate the relationship between diabetes management self-efficacy and depression, anxiety, emotional intelligence, and sleep quality; and (4) to investigate factors associated with diabetes management self-efficacy.

## Methods

Ethics statement: This study was approved by the Institutional Review Board of Chonnam National University (No. 1040198-180418-HR-023-03). A document substituting for an informed consent form was obtained from the participants online.

### Study design

This descriptive correlational study was designed to identify the factors affecting diabetes management self-efficacy in pregnant women with GDM. The description followed the STROBE (Strengthening the Reporting of Observational Studies in Epidemiology) reporting guidelines (https://www.strobe-statement.org/).

### Setting

To collect data, the researchers investigated online communities that primarily focus on pregnant women with GDM, and found that there was no such community on the Daum online portal and two communities on the Naver online portal. The questionnaires were collected from the GDM online communities that allowed us to post an enrollment notice. At the same time, the enrollment notices were posted on the authors’ blogs to facilitate participation through a Google online survey. Data were collected from July 19, 2018 to September 28, 2018.

Before answering the questionnaire, the participants voluntarily consented to participate in the study after going over a substitute consent form which described the purpose and method of the study and stipulated that: (1) a participant could withdraw from the study at any time if she did not want to participate, even after completing the survey, (2) the anonymity of personal information would be guaranteed, (3) the data would be used only for research purposes, and (4) the data would be retained for 3 years. Anonymity was maintained throughout the survey process and, after the survey was completed, a predetermined online gift certificate was provided to the participants by e-mail or text message.

### Participants

The accessible population of this study was pregnant women with GDM who joined and used online communities. Convenience sampling was conducted to recruit participants from two communities on the Naver online portal. The inclusion criteria were (1) pregnant women who were diagnosed with GDM after 20 weeks of gestation and used an online GDM community and (2) women who understood the purpose of this study, voluntarily consented to participate in the study, and submitted a document substituting for a consent form online. Women who had previously been diagnosed with type 1 or type 2 diabetes or were not pregnant at the time of the study were excluded.

### Study size

**Figure 1.**
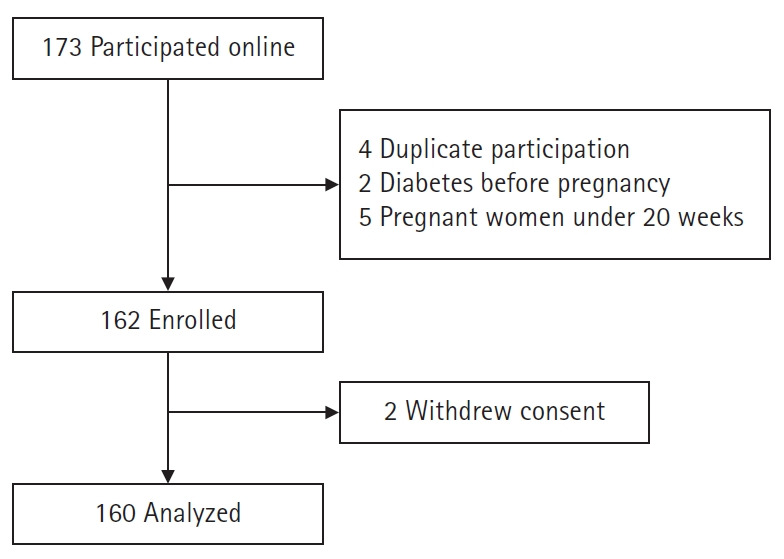
Flow diagram of participants.

Sample size calculation was done using G*Power 3.1.9.2 (HHU, Dusseldorf, Germany) with the significance level (α) set at .05, an effect size (f^2^) of .15, and a power (1-β) of .80 for multiple regression analysis involving 20 independent variables, which resulted in 157 participants. The effect size was determined based on the median effect size of f^2^=0.15 for multiple regression analysis according to the Cohen criteria [12]. The target population size was 173 people, considering a dropout rate of approximately 10% based on a previous study on pregnancy with GDM [13]. The online survey was closed when 173 questionnaires were received. A total of 160 samples were used in the final analysis, excluding four duplicated questionnaires, two questionnaires from pregnant women who had been diagnosed with diabetes mellitus before pregnancy, five questionnaires from pregnant women with less than 20 weeks of gestation, and two questionnaires from those who withdrew consent (Figure 1).

### Instruments

All study instruments were used with the authors’ permission.

#### Diabetes management self-efficacy

The modified Self-Efficacy for Diabetes Management tool adapted by Yeom [14] was used for the current study. The tool had originally been developed by the Stanford Patient Education Research Center [15]. It uses a 10-point Likert scale ranging from 1 point for “not at all confident” to 10 points for “very confident” and consists of a total of eight items. A higher score indicates a higher level of self-efficacy. The reliability of the tool, as shown by the Cronbach’s alpha coefficient, was 0.83 at the time of tool development [14] and 0.85 in the current study.

#### Depression

To measure the degree of depression in pregnant women, this study used the Edinburgh Postnatal Depression Scale, which was originally developed by Cox et al. [16] and adapted by Han et al. [17]. The tool uses a Likert scale (0-3 points) and has a total of 10 items, and a higher score indicates a higher degree of depression. The reliability of the tool, as shown by the Cronbach’s alpha coefficient, was 0.83 at the time of development [17] and 0.87 in the current study.

#### Anxiety

This study used the State-Trait Anxiety Inventory (STAI)-X, which was developed by Spielberger [18] and adapted by Han et al. [19]. This tool uses a 4-point Likert scale (1 point for “not at all” to 4 points for “very much”) and consists of a total of 40 items (20 items for state anxiety and 20 items for trait anxiety), but this study used only the state anxiety scale which measures the level of current anxiety. At the time of tool development, the reliability of the Korean version of the STAI, as shown by the Cronbach’s alpha coefficient, was 0.93 [19], and it was 0.93 in the current study.

#### Emotional intelligence

The Emotional Intelligence Scale (EIS), which was developed by Wong and Law [20] and was adapted by Jung [21], was used in this study. The EIS has a total of 16 items and consists of four subdomains: self-emotion appraisal, emotion appraisal of others, regulation of emotions, and use of emotions. It uses a 7-point Likert scale ranging from 1 point for “not at all” to 7 points for “strongly agree,” with higher scores indicating higher emotional intelligence. The reliability of the original tool, as shown by the Cronbach’s alpha coefficient was 0.87 [21], and it was 0.92 in the current study.

#### Sleep quality

The Verran & Snyder-Halpern Sleep Scale, which had been developed by Snyder-Halpern and Verran [22] and was adapted by Kim and Kang [23], was used in this study. It consists of a total of eight items and uses a visual analog scale ranging from 0 to 10. A higher score indicates better sleep quality. Reliability of the original tool shown by the Cronbach’s alpha coefficient was 0.82 [23] and it was 0.73 in the current study.

#### General and clinical characteristics

The general characteristics and clinical characteristics of the participants were evaluated based on previous studies [13]. General characteristics included age, gestational age, education, religion, number of children, occupation, and economic status. Clinical characteristics included duration of management of GDM, pre-pregnancy body mass index (BMI), past history of GDM, family history of GDM or diabetes, experience of GDM education, and blood sugar management methods used for GDM including regular glucose checks, diet, exercise, and insulin treatment.

### Data analysis

Descriptive statistics were utilized to analyze the participants’ general and clinical characteristics, depression, anxiety, emotional intelligence, sleep quality, and diabetes management self-efficacy. The differences in diabetes management self-efficacy according to the participants’ general and clinical characteristics were analyzed using the independent t-test, and Pearson correlation coefficients were used to determine the relationship between anxiety, emotional intelligence, sleep quality, and diabetes management self-efficacy. For factors affecting the diabetes management self-efficacy among the pregnant women with GDM, multiple regression analysis with the enter method was performed, and the normal distribution of continuous variables was confirmed by the Kolmogorov-Smirnov test. IBM SPSS ver. 24.0 (IBM Corp., Armonk, NY, USA) was used for the data analysis.

## Results

### General and clinical characteristics of participants

The mean age of the 160 pregnant women with GDM who participated in this study was 33.21±3.15 years (range, 26–43 years) and the mean number of weeks of gestation was 29.46±3.98 weeks (range, 21–40 weeks). The number of participants with a college degree or higher (n=139, 86.9%) was greater than those who had a high school diploma or less (n=21, 13.1%). Eighty-eight women (55.0%) were religious while 72 (45.0%) were non-religious. More participants had no child (n=109, 68.1%) than those who had at least one child (n=51, 31.9%), and a greater number of women had a job (n=94, 58.8%) than those who had no job (n=66, 41.3%). The majority of participants (n=143, 89.4%) responded that their perceived financial status was below average (Table 1).

For clinical characteristics, the average number of days of GDM management since being diagnosed with GDM was 37.57±29.78 days (range, 0–149 days), and the average pre-pregnancy BMI was 23.19±4.41 kg/m^2^ (range, 15.43–37.71 kg/m^2^). Of the total participants, 12 (7.5%) had a history of GDM and 59 (36.9%) had a family history of diabetes or GDM. There were 103 patients (64.4%) who had participated in GDM education. The GDM management methods included regular glucose checks (90.6%), dietary management (96.3%), and exercise (69.4%), and 21 participants (13.1%) underwent insulin treatment (Table 1).

### Depression, anxiety, emotional intelligence, sleep quality, and diabetes management self-efficacy of the participants

The average depression score of the study participants was 10.36±5.30 points (51.9% of the participants were in the high-risk group with 10 points or less), and the average score for anxiety was 41.65±11.16 points. The average score for emotional intelligence was 78.04±13.70 points. For each subdomain the scores were as follows: self-emotion appraisal, 20.94±3.91 points; emotion appraisal of others, 19.81±3.15 points; use of emotions, 18.16±4.28 points; and regulation of emotions, 18.98±4.24 points. The average score for sleep quality was 42.01±10.09 points, and that for diabetes management self-efficacy was 52.29±12.38 points (Table 2).

### Differences in diabetes management self-efficacy according to the general and clinical characteristics of participants

Analysis of the differences in diabetes management self-efficacy according to the general characteristics of the study participants (Table 1) showed that those with a college degree or higher had significantly higher diabetes management self-efficacy than those who had a high school diploma or below (t=2.60, *p*=.010). An analysis of the differences in diabetes management self-efficacy according to the clinical characteristics of the study participants (Table 1) showed that exercise was associated with significant differences in self-efficacy in diabetes management; those who did exercise had a significantly higher diabetes management self-efficacy than those who did not (t=–2.04, *p*=.043).

### Relationships among depression, anxiety, emotional intelligence, sleep quality, and diabetes management self-efficacy of participants

The results of analyzing the relationship between depression, anxiety, emotional intelligence, sleep quality, and diabetes management self-efficacy of the study participants are as follows (Table 3): the diabetes management self-efficacy score showed negative correlations with depression (r=–.18, *p*=.022) and anxiety (r=–.25, *p*=.001), whereas it showed positive correlations with emotional intelligence (r=.56, *p*<.001) and sleep quality (r=.34, *p*<.001).

### Factors affecting diabetes management self-efficacy

The results of stepwise multiple regression analysis performed to identify factors affecting the self-efficacy of diabetes management in pregnant women with GDM are presented in Table 4. To determine the factors affecting diabetes management self-efficacy, characteristics that showed significant differences, including educational background (1, university degree or higher) and exercise (1, yes), were treated as dummy variables and entered as independent variables along with depression, anxiety, emotional intelligence, and sleep quality for the multiple regression analysis.

One data point that was an outlier (absolute standardized residual value of 3 or more) was removed, and then multiple regression analysis was performed on 159 people. In the examination of autocorrelation (independence) of the errors, the Durbin Watson statistic was close to 2 (specifically, 1.90), indicating there was no autocorrelation between the error terms. In the multicollinearity test, the range of tolerance was >0.1 (specifically, 0.33–0.99) and the variance inflation factor was also ≤10 (specifically, 1.01–3.02), indicating there was no problem of multicollinearity. In addition, the assumptions of linearity, normality, and equal variance of residuals were met, and the Cook distance value for detecting outliers did not exceed 1.0, showing there were no outliers.

As a result of the multiple regression analysis, the diabetes management self-efficacy model for pregnant women with GDM showed a statistical significance (F=17.58, *p*<.001) with an explanatory power of 38.6%. Evaluation of the relative importance of the input variables showed emotional intelligence (β=.47, *p*<.001) was followed by sleep quality (β=.23, *p*=.001) and exercise (β=.18, *p*=.005).

## Discussion

Due to the absence of previous studies that investigated diabetes management self-efficacy and emotional intelligence among pregnant women with GDM, it was difficult to compare the findings of this study directly to those of other studies. However, a previous study [8], which suggested that emotional intelligence is a personal resource for facilitating lifelong diabetes management in type 1 diabetes patients, demonstrated the positive effects of emotional intelligence on diabetes management. This study was comparable to the current study in that it brought the concept of emotional intelligence (i.e., an individual’s ability to deal with emotional problems) to the surface, based on the observation of a similar group of patients who had difficulty in daily health management due to abnormal blood sugar problems. In addition to the maternal and fetal health status examinations and diabetes management performed in clinical settings, a program that includes interventions for improving patients’ emotional factors, especially emotional intelligence, is needed in order to significantly increase diabetes management self-efficacy. Sleep quality, which was found to have a significant influence on the self-efficacy of diabetes management in the pregnant women with GDM in this study, has not been addressed in previous research. However, considering the results of a systematic review showing that poor sleep quality increased the risk of GDM [10] and this study, which found that sleep quality was a factor that affects positive diabetes management self-efficacy for pregnant women with GDM, strategies to improve sleep quality should be developed.

It is necessary to improve diabetes management self-efficacy by developing a program that considers emotional intelligence, sleep quality, and exercise so that pregnant women with GDM can recognize their emotions, express negative emotions, and find support in the diabetes management process.

Conversely, results of the multiple regression analysis showed that depression and anxiety were not significant influencing factors for diabetes management self-efficacy. However, in a study by Jeong [24], depression was a predictor of self-efficacy in pregnant women with GDM, and a higher level of depression was associated with a lower level of self-efficacy. In a large cohort study in Canada [25], women with a history of anxiety disorder had a slightly increased risk of developing GDM. In view of these results, further research on the effects of depression and anxiety on pregnant women with GDM is warranted. Self-efficacy in diabetes management needs to be further elucidated through continuous research that considers related factors such as depression and anxiety. This study is significant in that it suggested diabetes management self-efficacy for women with GDM can be improved by considering factors such as emotional intelligence, sleep quality, and exercise, rather than attributing it to a negative emotional state (depression and anxiety) as the sole significant influencing factor.

Educational background and exercise levels were statistically significant factors related to the level of diabetes management self-efficacy according to the general and clinical characteristics of the participants of this study; specifically, those who had a college degree or higher and those who exercised had higher diabetes management self-efficacy scores. Although few studies have directly analyzed subjects’ educational background in relation to their diabetes management self-efficacy, one previous study [26] showed that highly educated people had higher diabetes management self-efficacy, which is consistent with the finding of this study. Further research is needed regarding the diabetes management self-efficacy among pregnant women with GDM in relation to educational background. In the current study, 69.4% of the respondents reported that they managed GDM through exercise, which is higher than 42% of people who exercised regularly in a previous study [13]. Unlike the previous study [13] that assessed the regularity of exercise, this study investigated whether the participants performed exercise or not, regardless of regularity. Therefore, people who exercised irregularly could have responded that they engaged in exercise, explaining the relatively higher percentage of people exercising in the current study.

In this study, depression and anxiety were inversely correlated with diabetes management self-efficacy, and emotional intelligence and sleep quality were positively correlated with diabetes management self-efficacy. The depression score of the pregnant women with GDM was 10.36 in the current study. In a previous study [27] that used the same tool to measure depression in pregnant women with GDM, the score was 6.45, indicating higher scores among the respondents of this study. Because the participants in this study consisted of pregnant women who used online communities for women with GDM, building a new relationship with strangers on the internet could have increased their level of depression [28], explaining the relatively higher depression score. In the current study, the anxiety score for pregnant women with GDM was 41.65 points out of a total score of 80 points. In a previous study [27] that used the same tool, pregnant women without GDM scored 34.44 points, and pregnant women with GDM scored 36.98 points; therefore, the anxiety score of pregnant women with GDM was relatively high in that study as well as in this study. The emotional intelligence score for pregnant women with GDM was 78.04 points out of a total score of 112 points, and the average score for each subdomain (total score of 28 points) was 20.94 for self-emotion appraisal, 19.81 points for emotion appraisal of others, 18.16 points for use of emotions, and 18.98 points for emotion regulation. Although the same tool was not used, a previous study [13] investigated emotional intelligence of pregnant women with GDM, and the emotional intelligence score was 38.27 points out of a total score of 55 points, which is similar to the result of this study. In the current study, the sleep quality score was 42.01 points out of a total of 80 points. In a study using the same tool [29], pregnant women without GDM scored 45.97 points while pregnant women with GDM scored 43.92 points (similar to this study), but there was no significant difference between the non-GDM group and the GDM group [29]. Since pregnancy itself affects sleep quality, long-term observation is needed to confirm the effect of GDM on sleep. In this study, the diabetes management self-efficacy score of the participants was 52.29 points out of a total of 80 points, which was above the intermediate level. Although the same tool was not used, a previous study [30] reported that the diabetes management self-efficacy score of pregnant women with GDM averaged 66.72 points out of a total of 100 points, also higher than the intermediate level.

The results of multiple regression analysis showed that predictive variables for diabetes management self-efficacy in pregnant women with GDM were emotional intelligence, sleep quality, and exercise. In other words, a higher level of emotional intelligence, higher sleep quality, and more exercise were associated with greater diabetes management self-efficacy.

A limitation of this study is that it was based on cross-sectional data obtained only from pregnant women with GDM using online communities, which may reflect sampling bias of women who are active. Another limitation is that although postpartum depression was measured, the study did not include physical symptoms of the participants. However, while many studies used depression, anxiety, and self-efficacy as independent variables to verify the effectiveness of programs for pregnant women with GDM, few studies have demonstrated negative emotions as influencing factors on diabetes management self-efficacy. In addition, this study provides basic data on individual factors that affect diabetes management self-efficacy in pregnant women with GDM, unlike most previous studies that investigated diabetes management self-efficacy as a factor influencing diabetes management outcomes.

In conclusion, to improve diabetes management self-efficacy in pregnant women with GDM, it is necessary to apply methods that can improve both physical and emotional health. Specifically, strategies should be developed to enhance emotional intelligence, sleep quality, and exercise. Furthermore, as the causal relationship between the major variables affecting diabetes management self-efficacy may be bidirectional, not unidirectional, future studies that explore a research model that verifies the direction of causality among the influencing variables would be beneficial.

## Figures and Tables

**Table 1.** Differences in diabetes management self-efficacy according to the general characteristics of participants (N=160)

Variable	Categories	Value	DM management self-efficacy		
			Mean±SD	t	*p*
*General characteristics*					
Age (year)		33.21±3.15 (range, 26–43)			
Gestational age (week)		29.46±3.98 (range, 21–40)			
Education	≤High school	21 (13.1)	45.86±12.20	–2.60	.010
	≥College	139 (86.9)	53.26±12.16		
Religion	No	88 (55.0)	51.99±12.92	–0.34	.737
	Yes	72 (45.0)	52.65±11.77		
Child(ren)	No	109 (68.1)	52.59±12.88	0.45	.656
	Yes	51 (31.9)	51.65±11.33		
Occupation	No	66 (41.2)	51.45±12.51	–0.71	.478
	Yes	94 (58.8)	52.87±12.32		
Perceived economic status	≤Below average	143 (89.4)	51.91±12.45	–1.12	.263
	≥High	17 (10.6)	55.47±11.66		
*Clinical characteristics*					
Duration of GDM management (week)		37.57±29.78 (range, 0-149)			
Pre-pregnancy BMI (kg/m^2^)		23.19±4.41 (range, 15.43-37.71)			
GDM history	No	148 (92.5)	52.22±12.60	–0.23	.818
	Yes	12 (7.5)	53.08±9.64		
DM or GDM family history	No	101 (63.1)	53.31±11.73	1.37	.174
	Yes	59 (36.9)	50.54±13.34		
Education on GDM	No	103 (64.4)	51.33±12.57	–1.32	.189
	Yes	57 (35.6)	54.02±11.96		
Regular glucose checks	No	15 (9.4)	50.00±15.39	–0.75	.454
	Yes	145 (90.6)	52.52±12.07		
Dietary management	No	6 (3.7)	43.33±20.76	–1.09	.324
	Yes	154 (96.3)	52.64±11.91		
Exercise	No	49 (30.6)	49.31±12.86	–2.04	.043
	Yes	111 (69.4)	53.60±11.99		
Treatment with insulin	No	139 (86.9)	52.85±12.81	1.48	.141
	Yes	21 (13.1)	48.57±8.39		

Middle: The economic level perceived by the subject is below average; High: the economic level perceived by the subject is above average. BMI: Body mass index; DM: diabetes mellitus; GDM: gestational diabetes mellitus.

**Table 2.** Descriptive statistics of research variables in participants (N=160)

Variable	Mean±SD	Minimum	Maximum	Possible range
Depression	10.36±5.30	1	25	0–30
Anxiety	41.65±11.16	20	70	20–80
Emotional intelligence	78.04±13.70	27	107	16–112
Self-emotion appraisal	20.94±3.91	7	28	7–28
Emotion appraisal of others	19.81±3.15	12	26	7–28
Use of emotions	18.16±4.28	4	27	7–28
Regulation of emotions	18.98±4.24	4	28	7–28
Sleep quality	42.01±10.09	17	69	0–80
Diabetes management self-efficacy	52.29±12.38	14	80	8-80

**Table 3.** Relationships among research variables (N=160)

Variable	r (*p*)			
	Depression	Anxiety	Emotional intelligence	Sleep quality
Depression	1			
Anxiety	.80 (<.001)	1		
Emotional intelligence	–.32 (<.001)	–.41 (<.001)	1	
Sleep quality	–.09 (.250)	–.19 (.019)	.28 (<.001)	1
Diabetes management self-efficacy	–.18 (.022)	–.25 (.001)	.56 (<.001)	.34 (<.001)

**Table 4.** Factors predicting diabetes management self-efficacy in pregnant women with gestational diabetes mellitus (N=159)

Variable	B	SE	β	t	*p*
(Constant)	2.96	7.38		0.40	.689
Education^†^	4.05	2.34	.11	1.75	.086
Exercise^†^	4.70	1.66	.18	2.83	.005
Depression	0.13	0.24	.06	0.53	.594
Anxiety	–0.07	0.12	–.07	–0.62	.536
Emotional intelligence	0.42	0.06	.47	6.58	<.001
Sleep quality	0.27	0.08	.23	3.39	.001
	Adjusted R^2^=38.6%, F=17.58, *p*<.001				

† Dummy variable references were education (≤ high school) and exercise (no).
